# Comparison of Genetic Profiles of Neonates in Intensive Care Units Conceived With or Without Assisted Reproductive Technology

**DOI:** 10.1001/jamanetworkopen.2023.6537

**Published:** 2023-04-04

**Authors:** Zhongwen Huang, Feifan Xiao, Hui Xiao, Yulan Lu, Lin Yang, Deyi Zhuang, Liping Chen, Qiufen Wei, Yinmo Jiang, Gang Li, Bingbing Wu, Zhiwei Liu, Wenhao Zhou, Huijun Wang

**Affiliations:** 1 Center for Molecular Medicine, Children’s Hospital of Fudan University, National Children’s Medical Center, Shanghai, China; 2 Department of Endocrinology and Inherited Metabolic Diseases, Children’s Hospital of Fudan University, National Children’s Medical Center, Shanghai, China; 3 Department of Neonatology, Xiamen Children’s Hospital, Xiamen, Fujian, China; 4 Department of Neonatology, Jiangxi Provincial Children’s Hospital, Nanchang, Jiangxi, China; 5 Department of Neonatology, Maternal and Child Health Care Hospital of Guangxi Zhuang Autonomous Region, Nanning, Guangxi, China; 6 Department of Neonatology, International Peace Maternity and Children Hospital of China Welfare Institution, School of Medicine, Shanghai Jiao Tong University, Shanghai, China; 7 Department of Neonatology, Children’s Hospital of Fudan University, National Children’s Medical Center, Key Laboratory of Neonatal Diseases, Ministry of Health, Shanghai, China

## Abstract

**Importance:**

A growing number of children are conceived with assisted reproductive technology (ART). However, there is a lack of studies systematically analyzing the genetic landscape of live-born children conceived through ART who need intensive care in the neonatal period.

**Objective:**

To investigate the incidence and type of molecular defects among neonates conceived through ART who are in intensive care units (NICUs) with suspected genetic conditions.

**Design, Setting, and Participants:**

This was a cross-sectional study using data from the China Neonatal Genomes Project, a multicenter national neonatal genome data set managed by the Children’s Hospital of Fudan University. All participants were from level III and IV NICUs and included 535 neonates conceived through ART with suspected genetic conditions, with data collected between August 1, 2016, and December 31, 2021, and 1316 naturally conceived neonates with suspected genetic conditions in the same clinical settings, with data collected between August 1, 2016, and December 31, 2018. The data were analyzed between September 2021 and January 2023.

**Exposures:**

Whole-exome sequencing or target clinical exome sequencing with pathogenic or likely pathogenic single-nucleotide variant (SNV) and copy number variation (CNV) detection was performed for each individual.

**Main Outcomes and Measures:**

The primary outcome was the molecular diagnostic yield, mode of inheritance, spectrum of genetic events, and incidence of de novo variants.

**Results:**

A total of 535 neonates conceived through ART (319 boys [59.6%]) and 1316 naturally conceived neonates (772 boys [58.7%]) were included. A genetic diagnosis was established for 54 patients conceived through ART (10.1%), including 34 patients with SNVs (63.0%) and 20 with CNVs (37.0%). In the non-ART group, 174 patients (13.2%) received a genetic diagnosis, including 120 patients with SNVs (69.0%) and 54 with CNVs (31.0%). The overall diagnostic yield was comparable between the ART group and the naturally conceived neonates (10.1% vs 13.2%; odds ratio [OR], 0.74; 95% CI, 0.53-1.02), as was the proportion of SNVs (63.0% vs 69.0%; OR, 0.68; 95% CI, 0.46-1.00) and CNVs (37.0% vs 31.0%; OR, 0.91; 95% CI, 0.54-1.53) detected by sequencing. Furthermore, the proportions of de novo variants in the ART group and the non-ART group were similar (75.9% [41 of 54] vs 64.4% [112 of 174]; OR, 0.89; 95% CI, 0.62-1.30).

**Conclusions and Relevance:**

This cross-sectional study of neonates in NICUs suggests that the overall genetic diagnostic yield and the incidence of de novo variants were similar between live-born neonates conceived through ART and naturally conceived neonates in the same settings.

## Key Points

**Question:**

Are molecular defects more common in newborns in the neonatal intensive care unit (NICU) conceived by assisted reproductive technology (ART) than in naturally conceived neonates?

**Findings:**

In this cross-sectional analysis of infants in NICUs for whom genetic analysis was completed, there was no significant difference in diagnoses of genetic conditions between those conceived with ART and those conceived unassisted (10.1% vs 13.2%). The proportion of de novo variants was also not significantly different.

**Meaning:**

This study suggests that in the NICU, the overall molecular diagnostic yield and the incidence of de novo variants were similar between live-born neonates conceived with ART and naturally conceived neonates.

## Teaser

This cross-sectional study investigates the incidence and type of molecular defects among neonates in neonatal intensive care units (NICUs) conceived with assisted reproductive technology (ART) compared with those conceived naturally.

## Introduction

Since the first infant conceived with in vitro fertilization (IVF) was born more than 40 years ago in the UK, the number of individuals conceived through assisted reproductive technology (ART) worldwide has increased much faster than expected.^1^ Advances in ART have made it possible for infertile families to have children while supporting demographic growth.^2^ In mainland China, ART cycles exceeded 1 million in 2016 and reached 1.15 million in 2017; this has resulted in an excess of 300 000 ART-conceived infants born each year.^3,4^ This number is expected to continue increasing over time.

Although ART is considered well established, the health of children conceived through ART remains a global concern.^5^ Previous studies have shown an increased risk of adverse perinatal outcomes, birth defects, developmental disorders, epigenetic alterations, and childhood cancer among children conceived through ART compared with children conceived spontaneously.^6,7,8,9,10,11,12,13^ A recently published Asian study found that among newborns conceived via ART, those admitted to the neonatal intensive care unit (NICU) were at high risk of infantile mortality, which may be attributed to a high prevalence of prematurity and congenital malformations.^14^ Previous studies have indicated that genetic causes were a leading contributor to neonatal morbidity and mortality among neonates in the NICU.^15,16,17^ Meanwhile, research on the incidence of de novo chromosomal microdeletions or specific point variants suggests that they are more common among individuals conceived through ART.^18,19,20,21,22^ However, the results were divergent, which may be because of small sample size, varied sample type, and different selections of population.

Overall, the genetic profile of newborns conceived through ART is largely unknown. In particular, with the widespread application of gene sequencing, there is a gap comparing the rate of genetic diagnosis in the NICU setting among children conceived through ART vs that of children conceived naturally. Studies in this field are still limited, and the research data on this population are lacking.

The goal of this study was to investigate the prevalence and type of molecular defects detected in a large Chinese cohort of ill neonates conceived through ART as well as among those conceived naturally.

## Methods

### Study Design, Setting, and Patient Population

This retrospective, cross-sectional study is part of the China Neonatal Genomes Project (CNGP), which manages a sizable neonatal genome data set containing genetic and clinical resources of 31 participating medical centers nationwide.^23^ The children were recruited from levels III and IV in the NICU at Children’s Hospital of Fudan University and other participating units, including children and maternal hospitals, pediatric departments from general hospitals, and children’s hospitals from other provinces or autonomous regions in mainland China. Furthermore, those with postnatal phenotypes suggestive of potential genetic causes were evaluated by experienced physicians and referred to our clinical genetics laboratory for genetic testing. As the leading unit of the CNGP, Children’s Hospital of Fudan University was responsible for genetic testing and data analysis. Written informed consent to participate in this study was provided by the participants’ legal guardians. Permission to perform this study was obtained by the ethics committee of the Children’s Hospital of Fudan University. This study followed the Strengthening the Reporting of Observational Studies in Epidemiology (STROBE) reporting guideline.

The inclusion criteria were (1) live-born neonate; (2) patients had 1 or more of the following clinical manifestations: congenital malformation, dysmorphia, metabolic abnormalities, recurrent infection, failure to thrive, multisystem anomalies, and neuromuscular anomaly; and (3) a minimum of 1 mL of venous blood sample from the patients. The exclusion criteria were (1) DNA quality control failed, (2) genetic diagnoses were received prenatally, and (3) families declined to participate in genetic testing or refused to follow up.

Two groups were classified according to the mode of conception. Assisted reproductive technology conception was defined as interventions that included in vitro–assisted hatching of human oocytes and sperm or embryos for reproduction purposes, which mostly included IVF and intracytoplasmic sperm injection (ICSI). Non-ART conception was defined as conceiving naturally. Enrollment of neonates conceived through ART occurred from August 1, 2016, to December 31, 2021. As a control, the non-ART group was randomly sampled at a ratio of 1:3 from the CNGP data set between August 1, 2016, and December 31, 2018. This control data set was from the same clinical settings with detailed clinical and genetic information that was published previously.^24^ The clinical information was obtained from electronic medical records and telephone call follow-ups.

### DNA Extraction and Sequencing

Peripheral blood samples were collected, and genomic DNA was extracted using the QIAamp DNA Blood Mini Kit (Qiagen) according to the manufacturer’s instructions. DNA fragments were enriched for clinical exome sequencing using the ClearSeq Inherited Disease panel kit (Agilent Technologies Inc), which covered 2742 genes, or whole-exome sequencing (WES) using the SureSelect XT Human All Exon V5 kit (Agilent Technologies Inc). Sequencing was performed on a HiSeq 2500, HiSeq X10, or NovaSeq 6000 platform (Illumina Inc). The test method was based on the test year and decided by the physicians’ and parents’ choice.

### Candidate Variation

Sequencing reads were aligned to the reference human genome assembly (NCBI build 37/hg19), using the Burrows-Wheeler Aligner, version 0.7.15-r1140. ANNOVAR, version 2019-10-24 (ANNOVAR), and Variant Effect Predictor, version 104.2 (Ensembl), were used for annotation. Calling and validation of single-nucleotide variants (SNVs) and copy number variations (CNVs) were performed using our in-house established multistep approach as described previously.^25,26^ The pathogenic class of variants was curated based on the criteria of the American College of Medical Genetics and Genomics.^27^ A variant was classified as pathogenic or likely pathogenic if the variant could explain the indication for testing and may be responsible for the patient’s clinical manifestation.

### Statistical Analysis

Data were analyzed from September 2021 to January 2023 using SPSS, version 20.0 software (IBM Corp). Categorical variables are presented mainly as frequency counts and proportions and compared with the χ^2^ test. For continuous variable group comparisons, the 2-sample *t* test and the 2-sample proportion test were used. A threshold of *P* < .05 with odds ratio (OR) and 95% CI was considered to be statistically significant.

## Results

### General Characteristics of the Study Population

A total of 1851 individuals (median age at genetic testing, 17.0 days [IQR, 6.0-30.0 days]) were included in the study. Of the 535 patients in the ART group, 319 (59.6%) were boys and 216 (40.4%) were girls; of the 1316 patients in the non-ART group, 772 (58.7%) were boys and 544 (41.3%) were girls (Table 1). The study flowchart is shown in the Figure, and the baseline characteristics of the 2 groups are shown in Table 1. Compared with the non-ART group, the rates of preterm birth (68.2% [365 of 535] vs 36.9% [486 of 1316]; OR, 3.89; 95% CI, 3.13-4.83), multiple births (58.9% [315 of 535] vs 26.5% [349 of 1316]; OR, 4.43; 95% CI, 3.57-5.50), and low birth weight (66.7% [357 of 535] vs 41.7% [549 of 1316]; OR, 2.94; 95% CI, 2.37-3.64) were significantly higher in the ART group.

**Table 1.** Clinical Characteristics of ART Group and Non-ART Group

Clinical characteristic	Patients, No. (%)				Difference in diagnosed ART cases and non-ART cases	
	ART group		Non-ART group			
	Total (n = 535)	Diagnosed (n = 54)	Total (n = 1316)	Diagnosed (n = 174)	OR (95% CI)	*P* value^a^
Sex						
Male	319 (59.6)	37 (68.5)	772 (58.7)	101 (58.0)	0.87 (0.58-1.30)	.50
Female	216 (40.4)	17 (31.5)	544 (41.3)	73 (42.0)	0.55 (0.32-0.96)	.03
Prematurity						
Yes	365 (68.2)	27 (50.0)	486 (36.9)	27 (15.5)	1.36 (0.78-2.36)	.28
>32-37 wk gestation	186 (34.8)	22 (40.7)	350 (26.6)	26 (14.9)	1.67 (0.92-3.04)	.09
28-32 wk gestation	113 (21.1)	5 (9.3)	148 (11.2)	1 (0.6)	6.81 (0.78-59.09)	.11
<28 wk gestation	67 (12.5)	0	7 (0.5)	0	NA	NA
No	159 (29.7)	27 (50.0)	823 (62.5)	145 (83.3)	0.96 (0.61-1.50)	.85
Missing data	11 (2.1)	0	7 (0.5)	2 (1.1)	NA	NA
Multiplicity						
Yes	315 (58.9)	22 (40.7)	349 (26.5)	5 (2.9)	5.17 (1.93-13.81)	<.001
No	194 (36.3)	32 (59.3)	952 (72.3)	154 (88.5)	1.02 (0.68-1.55)	.91
Missing data	26 (4.9)	0	15 (1.1)	15 (8.6)	NA	NA
Low birth weight						
Yes	357 (66.7)	26 (48.1)	549 (41.7)	33 (19.0)	1.23 (0.72-2.09)	.45
>1500-2500 g	201 (37.6)	20 (37.0)	373 (28.3)	31 (17.8)	1.22 (0.68-2.20)	.51
>1000-1500 g	115 (21.5)	4 (7.4)	138 (10.5)	2 (1.1)	2.45 (0.44-13.63)	.52
<1000 g	41 (7.7)	1 (1.9)	38 (2.9)	0	NA	NA
No	167 (31.2)	29 (53.7)	755 (57.4)	133 (76.4)	0.98 (0.63-1.53)	.94
Missing data	11 (2.1)	0	12 (0.9)	8 (4.6)	NA	NA
Admission concern to NICU						
Prematurity and related complications	297 (55.5)	12 (22.2)	368 (28.0)	19 (10.9)	0.77 (0.37-1.62)	.50
Congenital anomalies	60 (11.2)	8 (14.8)	368 (28.0)	21 (12.1)	2.54 (1.07-6.04)	.03
Neonatal jaundice	54 (10.1)	11 (20.4)	106 (8.1)	13 (7.5)	1.83 (0.76-4.41)	.18
Respiratory problems	79 (14.8)	9 (16.7)	161 (12.2)	21 (12.1)	0.86 (0.37-1.97)	.72
Neonatal convulsions	19 (3.6)	7 (13.0)	139 (10.6)	30 (17.2)	2.12 (0.77-5.85)	.14

Abbreviations: ART, assisted reproductive technology; NA, not applicable; NICU, neonatal intensive care unit; OR, odds ratio.

^a^ Calculated using the χ^2^ test.

**Figure.**
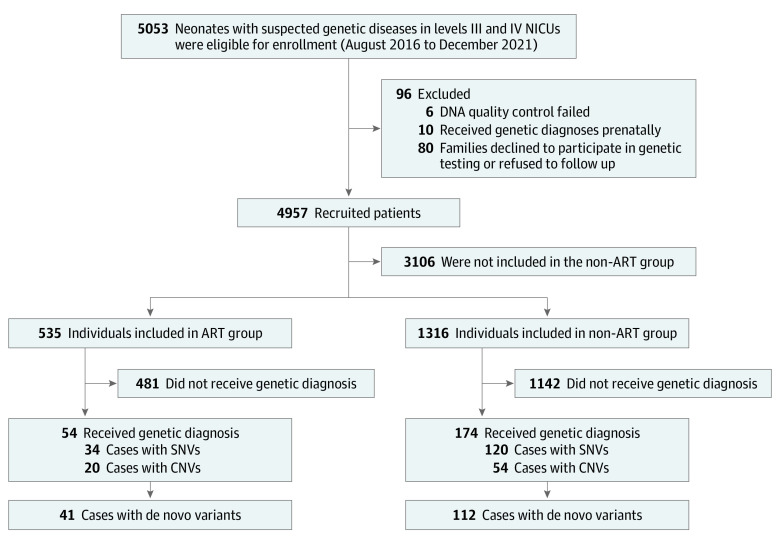
Study Flowchart The non–assisted reproductive technology (ART) group was randomly sampled at a ratio of 1:3 from the same clinical setting, with detailed clinical and genetic information. CNVs indicates copy number variations; NICUs, neonatal intensive care units; and SNVs, single-nucleotide variants.

The top admission concerns for patients in the NICU in the ART and non-ART groups were prematurity and related complications (55.5% [297 of 535] vs 28.0% [368 of 1316]; OR, 3.25; 95% CI, 2.61-3.96), congenital anomalies (11.2% [60 of 535] vs 28.0% [368 of 1316]; OR, 0.33; 95% CI, 0.24-0.44), respiratory problems (14.8% [79 of 535] vs 12.2% [161 of 1316]; OR, 1.24; 95% CI, 0.93-1.66) and neonatal convulsions (3.6% [19 of 535] vs 10.6% [139 of 1316]; OR, 0.31; 95% CI, 0.19-0.51) (Table 1).

Because the conditions of critically ill newborns are complicated and cannot be classified by a single symptom, we classified them according to the organ system involved to describe their clinical features further. The top 4 clinical features in the ART group were cardiovascular (67.9% [363 of 535]), hepatic (63.1% [338 of 535]), respiratory (60.2% [322 of 535]), and neuromuscular (41.3% [221 of 535]) (eTable 1 in Supplement 1). In the non-ART group, the most affected systems were neuromuscular (53.1% [699 of 1316]), followed by hepatic (51.0% [671 of 1316]), hematologic (45.4% [597 of 1316]), and metabolic (42.6% [560 of 1316]). These rankings differed when examining only patients who received a diagnosis. For example, respiratory phenotypes ranked second among those in the ART group who received a diagnosis (51.9% [28 of 54], while hematologic phenotypes ranked second among those in the non-ART group who received a diagnosis (29.3% [51 of 174]).

### Genetic Diagnosis Based on Exome Sequencing

In the ART cohort, 54 of 535 patients (10.1%) received genetic diagnoses that could explain their symptoms, of whom 63.0% (34 of 54) had SNVs (Table 2) and 37.0% (20 of 54) had CNVs (Table 3). The SNVs and CNVs found among these 54 patients were scattered across chromosomes (eFigure in Supplement 1). Of them, 21 SNVs and 20 CNVs were de novo, for a 75.9% (41 of 54) de novo variant (DNV) rate.

**Table 2.** Genetic Findings and Mode of Inheritance in 54 Diagnosed ART Cases and 174 Non-ART Cases (Single-Nucleotide Variants)

Single-nucleotide variant	ART cases diagnosed (54 of 535 [10.1%])			Non-ART cases diagnosed (174 of 1316 [13.2%])		
	No. of cases (34 of 54 [63.0%])	Gene	No. of de novo variants	No. of cases (120 of 174 [69.0%])	Gene	No. of de novo variants
Autosomal dominant	25	*KMT2D*, *KCNQ2* (in 2 patients), *TSC1*, *F11*, *JAG1*, *COL2A1* (in 2 patients), *ANKRD11*, *PTPN11* (in 2 patients), *SCN2A*, *ABCC8*, *NSD1*, *MAF* (in 2 patients), *NF1*, *MYH7*, *MYH9*, *RUNX2*, *CAMK2B*, *RAF1*, *CAMTA1*, *FGFR3*, and *BRAF*	18	73	*ABCC8* (in 5 patients), *ACTA1* (in 3 patients), *ANK1* (in 2 patients), *ASXL1*, *ASXL3*, *CHD7* (in 3 patients), *COL2A1* (in 4 patients), *COL7A1* (in 2 patients), *CRYGD*, *ELN*, *F11*, *FBN1*, *FGFR2*, *KCNJ11*, *KCNQ2* (in 14 patients), *KMT2A*, *KMT2D* (in 4 patients), *KRT1* (in 2 patients), *KRT10*, *MAP2K1*, *MYCN*, *NIPBL*, *NSD1* (in 3 patients), *PROS1*, *PTPN11* (in 3 patients), *RASA1*, *RPS19*, *SCN8A*, *SPTB*, *STXBP1*, *TCOF1*, *TP63*, *TRPV4*, *TSC2*, *TUBA1A*, *VWF*, *WT1*, and *ZEB2* (in 2 patients)	88
Autosomal recessive	4	*DUOX2*, *GCDH*, *COL7A1*, and *RYR1*	0	38	*ALDH7A1*(in 3 patients), *ALOX12B*, *BSND*, *COL7A1* (in 5 patients), *CRLF1*, *F5*, *FBP1*, *GCDH*, *GLDC*, *GNPTAB*, *IL10RA*, *IVD* (in 2 patients), *LAMC2*, *MMACHC* (in 4 patients), *MUT* (in 5 patients), *NCF1*, *PAH* (in 2 patients), *PCCB*, *PEX1*, *SCNN1A*, *SPINK5*, *TCIRG1*, and *TGM1*	0
X-linked dominant	3	*G6PD* (in 2 patients) and *USP9X*	2	0	NA	0
X-linked recessive	2	*ATP7A* and *DMD*	1	9	*AVPR2*, *F8*, *GATA1*, *GPC3*, *MTM1* (in 2 patients), and *OTC* (in 3 patients)	0

Abbreviations: ART, assisted reproductive technology; NA, not applicable.

**Table 3.** Genetic Findings and Mode of Inheritance in 54 Diagnosed ART Cases and 174 Non-ART Cases (CNVs)

CNV	ART cases diagnosed (54 of 535 [10.1%])			Non-ART cases diagnosed (174 of 1316 [13.2%])		
	No. of cases (20 of 54 [37.0%])	CNV size	No. of de novo variants	No. of cases (54 of 174 [31.0%])	CNV size	No. of de novo variants
DEL	12	>5 Mb in 2 patients; 1-5 Mb in 7 patients; <1 Mb in 3 patients	12	34	>5 Mb in 14 patients; 1-5 Mb in 16 patients; <1 Mb in 4 patients	34
DUP	5	>5 Mb in 2 patients; 1-5 Mb in 1 patient; <1 Mb in 2 patients	5	2	>5 Mb in 2 patients	2
DEL and DUP	1	DEL <1 Mb; DUP 1-5 Mb	1	0	NA	0
Aneuploidy	2	chrX, chr18	2	18	chr4, chr9, chr18 (in 4 patients), chr21 (in 10 patients), chrX (in 2 patients)	18

Abbreviations: ART, assisted reproductive technology; CNV, copy number variation; DEL, deletion; DUP, duplication; Mb, megabase; NA, not applicable.

In the non-ART cohort, 174 of 1316 patients (13.2%) harbored pathogenic or likely pathogenic variations, including 120 patients (69.0%) who were identified with SNVs (Table 2) and 54 patients (31.0%) with CNVs (Table 3). Among the 174 patients, 64.4% (112 of 174) had DNVs. There was no statistically significant difference in overall diagnostic yield (10.1% vs 13.2%; OR, 0.74; 95% CI, 0.53-1.02), DNV rate (75.9% vs 64.4%; OR, 0.89; 95% CI, 0.62-1.30), SNV rate (63.0% vs 69.0%; OR, 0.68; 95% CI, 0.46-1.00), or CNV rate (37.0% vs 31.0%; OR, 0.91; 95% CI, 0.54-1.53) between the 2 groups (Table 2 and Table 3; eTable 2 in Supplement 1).

### SNV Profiles Detected in the ART Group

Of the 34 patients in the ART group with SNVs, 39 allele variants were detected (4 patients had biallelic compound heterozygous variants): 5 frameshift, 7 nonsense, 2 splice, and 25 missense variants (Table 2; eTable 2 in Supplement 1). A total of 25 patients had an autosomal dominant inheritance pattern of disease (involving genes *KMT2D*, *KCNQ2* [2 patients], *TSC1*, *F11*, *JAG1*, *COL2A1* [2 patients], *ANKRD11*, *PTPN11* [2 patients], *NF1*, *SCN2A*, *ABCC8*, *NSD1*, *MAF* [2 patients], *MYH7*, *MYH9*, *RUNX2*, *CAMK2B*, *RAF1*, *CAMTA1*, *FGFR3*, and *BRAF*). As for the sources of variants, 7 patients inherited the variation from their parents, and 18 patients had DNVs. In addition, an autosomal recessive inheritance pattern of disease was detected for 4 patients (all involving biallelic compound heterozygous variants with *DUOX2*, *GCDH*, *COL7A1*, and *RYR1*), inheriting alleles with variants from each parent. Among the other 5 patients with X-linked genes (*ATP7A, DMD, G6PD,* and *USP9X*), 3 (2 boys and 1 girl) carried DNVs, and 2 (both boys) had maternally inherited variants (eTable 2 in Supplement 1).

### Chromosome Distribution of 20 CNVs in the ART Group

Next-generation sequencing database CNV analysis identified 20 patients with pathogenic or likely pathogenic de novo CNVs among patients in the ART group, encompassing 12 deletions, 5 duplications, 2 aneuploidies, and 1 case with both a deletion and a duplication (Table 3; eTable 2 in Supplement 1). Chromosomal deletions or duplications involved 22q11.21 del (DiGeorge syndrome), 15q11.2-15q13.2 dup (Prader-Willi syndrome), 4p16.3 del (Wolf-Hirschhorn syndrome), Xp21.1 del (Duchenne muscular dystrophy), and other regions (eFigure in Supplement 1).

### Perinatal Characteristics of 54 Patients in the ART Group Who Received a Diagnosis

The type of ART included 37 of 54 patients (68.5%) who underwent conventional IVF, in which oocytes are incubated with sperm in a dish and the male gamete fertilizes the oocyte naturally, and 17 patients (31.5%) who received ICSI, in which a single sperm is injected into an oocyte cytoplasm using a glass micropipette (Table 4). Both maternal and paternal age ranged from 24 to 45 years, with a median of 32 years (IQR, 29-35 years) and 33 years (IQR, 30-35.5 years), respectively. There were 38 of 54 cases (70.4%) in which the mother was aged 35 years or younger and 48 of 54 cases (88.9%) in which the father was aged 40 years or younger. The reasons for undergoing ART varied and can be roughly divided into the following situations: maternal factors (12 patients with fallopian tube problems, 5 with ovarian factors, 2 with endometriosis, and 1 with ovulation obstacle), paternal factors (16 patients with oligospermia and/or asthenospermia), both parental factors (2 patients), familial history of hereditary disease (1 patient), recurrent miscarriage history (11 patients), and unknown reasons (4 patients). Thirty-two neonates were singletons, and 22 were twins; 30 of the 54 neonates in the ART group (55.6%) were the product of first gestation. A total of 75.9% of mothers (41 of 54) reported uneventful pregnancies. Noninvasive prenatal testing (NIPT) was performed in 48 cases, of which 2 cases suggested abnormalities and 46 (95.8%) showed no abnormalities. Abnormal B-mode ultrasonographic findings were observed for 9 patients. Low-frequency fetal movement or polyhydramnios was reported for the other 2 cases.

**Table 4.** Perinatal Characteristics of 54 Diagnosed Neonates Conceived Via ART

Category	Diagnosed ART cases, No. (%)	No. of SNVs	No. of CNVs
Type of ART			
IVF^a^	37 (68.5)	25	12
ICSI	17 (31.5)	9	8
Maternal age, y			
Median (IQR) [range]	32 (29-35) [24-45]	NA	NA
≤35	38 (70.4)	24	14
>35	16 (29.6)	10	6
Paternal age, y			
Median (IQR) [range]	33 (30-35.5) [24-45]	NA	NA
≤40	48 (88.9)	30	18
>40	6 (11.1)	4	2
Reason for ART			
Factors			
Maternal^b^	20 (37.0)	12	8
Paternal^c^	16 (29.6)	9	7
Both parental infertility	2 (3.7)	1	1
Parental chromosome abnormalities	1 (1.9)	1	0
Recurrent miscarriage	11 (20.4)	8	3
Unknown reason^d^	4 (7.4)	4	0
Single or multiple births			
Singleton	32 (59.3)	18	14
Twin	22 (40.7)	16	6
Gestational order			
G1	30 (55.6)	20	10
G2	13 (24.1)	6	7
G3	6 (11.1)	4	2
More than G3	5 (9.3)	4	1
Prenatal examination results			
Normal	41 (75.9)	26	15
B-mode ultrasonography indicated structural malformations^e^	9 (16.7)	7	2
NIPT and/or amniocentesis suggest abnormalities^f^	2 (3.7)	0	2

Abbreviations: ART, assisted reproductive technology; CNV, copy number variation; ICSI, intracytoplasmic sperm injection; IVF, in vitro fertilization; NA, not applicable; NIPT, noninvasive prenatal testing; SNV, single-nucleotide variant.

^a^ One case conceived by preimplantation genetic testing has been counted as IVF.

^b^ Maternal factors included 12 cases with fallopian tube problems, 5 with ovarian factors, 2 with endometriosis, and 1 with ovulation obstacle.

^c^ Paternal factors included oligospermia and/or asthenospermia.

^d^ Parents could not figure out why they could not conceive naturally.

^e^ Included 1 case with abnormal B-mode ultrasonographic findings while amniocentesis and NIPT results were normal.

^f^ A total of 48 patients’ mothers underwent NIPT, and 46 of them showed normal results.

## Discussion

In this cross-sectional study, the incidence and type of molecular defects among neonates conceived through ART who are in NICUs with suspected genetic condition were investigated, and the genetic profiles of those conceived through ART were compared with the genetic profiles of those not conceived by ART. We observed no significant differences in genetic profiles between groups, which included the diagnosis rate and the percentage of SNVs and CNVs, nor in the proportion of DNVs between the 2 groups.

We did find a higher prevalence of prematurity, multiplicity, and low birth weight among neonates conceived through ART, which is consistent with other studies.^14,28,29^ However, to our knowledge, no study has been specifically designed to explore the perinatal outcomes of neonates conceived through ART compared with naturally conceived neonates in the NICU setting. In addition, the association of preterm birth, multiple births, and low birth weight with genetic causes among neonates conceived though ART remains unclear and requires further investigation.

De novo variants play an important role in human diseases and are primary targets in the analysis of rare genetic disorders.^30,31,32^ Given the nature of spontaneous human mutagenesis and the way in which it arises during early embryonic development, DNVs occur unavoidably.^33^ The natural course and frequency of DNVs have been assessed in several studies.^34,35,36^ However, to our knowledge, few studies have examined the associations of conception via ART and natural conception with overall DNVs. Our results are consistent with those of Zamani Esteki et al,^37^ in which de novo CNVs were present in 12 of the 111 families (10.8%), and are also in line with those of the study by Smits et al,^22^ which revealed no significant differences in the number of DNVs in 53 children and their parents. In contrast, another Chinese birth cohort study involving 365 families with genome sequencing data found more germline DNVs in children born after ART conception than non-ART conception.^38^ However, 98.2% of DNVs reported in that study were located in the noncoding regions of the genome. It is difficult to determine how those DNVs were associated with the phenotype, and the lack of children’s disease information limited the clinical significance of evaluating those DNVs.

De novo variants can occur at any stage, whether before fertilization in the germ cells or during the cleavage and blastocyst stages in early embryonic development.^37,39,40,41^ In our study, DNVs were detected among 2 sets of twins. Cases 11 and 12 are monochorionic diamniotic twins conceived via IVF, both of whom carried the same de novo pathogenic variant of *PTPN11*. Three possible speculations exist; one possibility is that the variant occurs as a result of a parental gonosomal variant or germline mosaicism, which affects the eggs or sperm; another is a de novo germline variant that occurs in a single germ and is transmitted to the child; and finally, the variant may have arisen postzygotically, from a very early stage in the fertilized eggs before the division and such that the variant presents in all cells in the 2 embryos. In another pair of twins (cases 21 and 22) who carried the same DNV of the *MAF* gene, 2 independent fertilized eggs were injected into the mother. Under such circumstances, the variant may originate from parental mosaicism. It is estimated that approximately 3% of germline DNVs originated as a mosaic in the germ cells of a parent.^39^ Such variants that can be transmitted to the next generation while undetectable in the parents’ blood are challenges for ART.

The similar molecular diagnosis rates in the ART and non-ART groups in the NICU suggest that ART status may not necessarily be a factor in determining whether perinatal genetic testing is offered. A negative prenatal testing result does not exclude the possibility of an unexpected variant. In our study, 95.8% of cases who underwent NIPT showed normal results even with abnormal ultrasonographic findings; amniocentesis karyotype analysis was performed for 3 cases, and only 1 was identified as euploidy. Recently, a study reported a 6.8% de novo chromosomal abnormality rate by prenatal amniocentesis karyotype analysis in fetuses conceived via IVF or ICSI, most of which were autosomal aneuploidy.^42^ However, routine NIPT, karyotype analysis, and chromosomal microarray have limitations in determining SNVs and small CNVs. In the prenatal setting, WES combined with chromosomal microarray has significantly improved the diagnosis rate of fetal genetic variation.^43,44^ However, rare variants remain difficult to detect without prenatal indications for WES.

### Limitations

This study has some limitations. First, it enrolled live-born children, while stillborn fetuses and terminations of pregnancy were not included. Therefore, this may lead to limitations in evaluating some fetal lethal SNVs or CNVs. Second, the data on the ART procedures of neonates conceived through ART who were undiagnosed were not collected in the CNGP. Third, many well-known potential risks, such as elder paternal age, unhealthy parental lifestyles, and infertility, were not examined.^34,45,46,47,48,49,50^ More extensive and prospective cohort studies are required to determine how these factors may be associated with ill neonates conceived with ART.

## Conclusions

This cross-sectional study of neonates in NICUs suggests that the overall diagnosis rate and the incidence of de novo SNVs or CNVs were similar between live-born neonates conceived through ART and naturally conceived neonates. This study will provide insight for physicians and couples considering ART.

## Notes

Supplement 1. **eTable 1.** The Ranking of Involved Organ Systems of the ART Group and Non-ART Group **eTable 2.** The Detailed Genetic Information of the 54 Diagnosed ART Cases **eFigure.** Distributions of Copy Number Variations (CNVs) and Single Nucleotide Variants (SNVs) in ART Offspring

Supplement 2.
Data Sharing Statement

## References

[1] Steptoe PC, Edwards RG. Birth after the reimplantation of a human embryo. Lancet. 1978;2(8085):366. doi:10.1016/S0140-6736(78)92957-4 79723

[2] Sunderam S, Kissin DM, Zhang Y, . Assisted reproductive technology surveillance—United States, 2018. MMWR Surveill Summ. 2022;71(4):1-19. doi:10.15585/mmwr.ss7104a1 35176012 PMC8865855

[3] Bai F, Wang DY, Fan YJ, . Assisted reproductive technology service availability, efficacy and safety in mainland China: 2016. Hum Reprod. 2020;35(2):446-452. doi:10.1093/humrep/dez245 32020190

[4] Qiao J, Wang Y, Li X, . A *Lancet* Commission on 70 years of women’s reproductive, maternal, newborn, child, and adolescent health in China. Lancet. 2021;397(10293):2497-2536. doi:10.1016/S0140-6736(20)32708-2 34043953

[5] Berntsen S, Söderström-Anttila V, Wennerholm UB, . The health of children conceived by ART: “the chicken or the egg?”. Hum Reprod Update. 2019;25(2):137-158. doi:10.1093/humupd/dmz001 30753453

[6] Boulet SL, Kirby RS, Reefhuis J, ; States Monitoring Assisted Reproductive Technology (SMART) Collaborative. Assisted reproductive technology and birth defects among liveborn infants in Florida, Massachusetts, and Michigan, 2000-2010. JAMA Pediatr. 2016;170(6):e154934. doi:10.1001/jamapediatrics.2015.4934 27043648 PMC4899282

[7] Zhang L, Zhang W, Xu H, Liu K. Birth defects surveillance after assisted reproductive technology in Beijing: a whole of population-based cohort study. BMJ Open. 2021;11(6):e044385. doi:10.1136/bmjopen-2020-044385 34162637 PMC8231031

[8] Luke B, Brown MB, Wantman E, . The risk of birth defects with conception by ART. Hum Reprod. 2021;36(1):116-129. doi:10.1093/humrep/deaa272 33251542 PMC8679367

[9] Hargreave M, Jensen A, Hansen MK, . Association between fertility treatment and cancer risk in children. JAMA. 2019;322(22):2203-2210. doi:10.1001/jama.2019.18037 31821431 PMC7081748

[10] Weng SS, Huang YT, Huang YT, Li YP, Chien LY. Assisted reproductive technology and risk of childhood cancers. JAMA Netw Open. 2022;5(8):e2230157. doi:10.1001/jamanetworkopen.2022.30157 36044210 PMC9434352

[11] Esteves SC, Roque M, Bedoschi G, Haahr T, Humaidan P. Intracytoplasmic sperm injection for male infertility and consequences for offspring. Nat Rev Urol. 2018;15(9):535-562. doi:10.1038/s41585-018-0051-8 29967387

[12] Wen SW, Miao Q, Taljaard M, . Associations of assisted reproductive technology and twin pregnancy with risk of congenital heart defects. JAMA Pediatr. 2020;174(5):446-454. doi:10.1001/jamapediatrics.2019.6096 32091547 PMC7042937

[13] Elhakeem A, Taylor AE, Inskip HM, ; Assisted Reproductive Technology and Future Health (ART-Health) Cohort Collaboration. Association of assisted reproductive technology with offspring growth and adiposity from infancy to early adulthood. JAMA Netw Open. 2022;5(7):e2222106. doi:10.1001/jamanetworkopen.2022.22106 35881399 PMC9327583

[14] Hii ZWS, Huang Z, Mathew JE, Lee LY. Retrospective analysis of neonates born after assisted reproductive technology and admitted to the neonatal intensive care unit. Ann Acad Med Singap. 2022;51(4):241-243. doi:10.47102/annals-acadmedsg.2021476 35506408

[15] French CE, Delon I, Dolling H, ; NIHR BioResource—Rare Disease; Next Generation Children Project. Whole genome sequencing reveals that genetic conditions are frequent in intensively ill children. Intensive Care Med. 2019;45(5):627-636. doi:10.1007/s00134-019-05552-x 30847515 PMC6483967

[16] Swaggart KA, Swarr DT, Tolusso LK, He H, Dawson DB, Suhrie KR. Making a genetic diagnosis in a level IV neonatal intensive care unit population: who, when, how, and at what cost? J Pediatr. 2019;213:211-217. doi:10.1016/j.jpeds.2019.05.054 31255390

[17] Raymond FL. Clinical genomics in critically ill infants and children. JAMA. 2020;323(24):2480-2482. doi:10.1001/jama.2020.8112 32573653

[18] Feng C, Wang LQ, Dong MY, Huang HF. Assisted reproductive technology may increase clinical mutation detection in male offspring. Fertil Steril. 2008;90(1):92-96. doi:10.1016/j.fertnstert.2007.06.004 18258231

[19] Zheng YM, Li L, Zhou LM, . Alterations in the frequency of trinucleotide repeat dynamic mutations in offspring conceived through assisted reproductive technology. Hum Reprod. 2013;28(9):2570-2580. doi:10.1093/humrep/det294 23861482

[20] Berntsen S, Laivuori H, la Cour Freiesleben N, . A systematic review and meta-analysis on the association between ICSI and chromosome abnormalities. Hum Reprod Update. 2021;27(5):801-847. doi:10.1093/humupd/dmab005 33956940

[21] Bonduelle M, Van Assche E, Joris H, . Prenatal testing in ICSI pregnancies: incidence of chromosomal anomalies in 1586 karyotypes and relation to sperm parameters. Hum Reprod. 2002;17(10):2600-2614. doi:10.1093/humrep/17.10.2600 12351536

[22] Smits RM, Xavier MJ, Oud MS, . De novo mutations in children born after medical assisted reproduction. Hum Reprod. 2022;37(6):1360-1369. doi:10.1093/humrep/deac068 35413117 PMC9156847

[23] Xiao F, Yan K, Wang H, . Protocol of the China Neonatal Genomes Project: an observational study about genetic testing on 100,000 neonates. Pediatr Med. 2021;4:28. doi:10.21037/pm-21-29

[24] Yang L, Wei Z, Chen X, . Use of medical exome sequencing for identification of underlying genetic defects in NICU: experience in a cohort of 2303 neonates in China. Clin Genet. 2022;101(1):101-109. doi:10.1111/cge.14075 34671977

[25] Yang L, Kong Y, Dong X, . Clinical and genetic spectrum of a large cohort of children with epilepsy in China. Genet Med. 2019;21(3):564-571. doi:10.1038/s41436-018-0091-8 29930392 PMC6681813

[26] Wang H, Xiao F, Dong X, . Diagnostic and clinical utility of next-generation sequencing in children born with multiple congenital anomalies in the China Neonatal Genomes Project. Hum Mutat. 2021;42(4):434-444. doi:10.1002/humu.24170 33502061

[27] Richards S, Aziz N, Bale S, ; ACMG Laboratory Quality Assurance Committee. Standards and guidelines for the interpretation of sequence variants: a joint consensus recommendation of the American College of Medical Genetics and Genomics and the Association for Molecular Pathology. Genet Med. 2015;17(5):405-424. doi:10.1038/gim.2015.30 25741868 PMC4544753

[28] Bănică AM, Popescu SD, Vlădăreanu S. Maternal and neonatal outcomes following in vitro fertilization: a cohort study in Romania. Exp Ther Med. 2022;23(1):34. doi:10.3892/etm.2021.10956 34824642 PMC8611488

[29] Blickstein I. Does assisted reproduction technology, per se, increase the risk of preterm birth? BJOG. 2006;113(suppl 3):68-71. doi:10.1111/j.1471-0528.2006.01126.x 17206968

[30] Goldmann JM, Wong WS, Pinelli M, . Parent-of-origin-specific signatures of de novo mutations. Nat Genet. 2016;48(8):935-939. doi:10.1038/ng.3597 27322544

[31] Veltman JA, Brunner HG. De novo mutations in human genetic disease. Nat Rev Genet. 2012;13(8):565-575. doi:10.1038/nrg3241 22805709

[32] Oud MS, Smits RM, Smith HE, ; Genetics of Male Infertility Initiative (GEMINI) Consortium. A de novo paradigm for male infertility. Nat Commun. 2022;13(1):154. doi:10.1038/s41467-021-27132-8 35013161 PMC8748898

[33] Goldmann JM, Veltman JA, Gilissen C. De novo mutations reflect development and aging of the human germline. Trends Genet. 2019;35(11):828-839. doi:10.1016/j.tig.2019.08.005 31610893

[34] Crow JF. The origins, patterns and implications of human spontaneous mutation. Nat Rev Genet. 2000;1(1):40-47. doi:10.1038/35049558 11262873

[35] Belyeu JR, Brand H, Wang H, . De novo structural mutation rates and gamete-of-origin biases revealed through genome sequencing of 2,396 families. Am J Hum Genet. 2021;108(4):597-607. doi:10.1016/j.ajhg.2021.02.012 33675682 PMC8059337

[36] Jónsson H, Sulem P, Arnadottir GA, . Multiple transmissions of de novo mutations in families. Nat Genet. 2018;50(12):1674-1680. doi:10.1038/s41588-018-0259-9 30397338

[37] Zamani Esteki M, Viltrop T, Tšuiko O, . In vitro fertilization does not increase the incidence of de novo copy number alterations in fetal and placental lineages. Nat Med. 2019;25(11):1699-1705. doi:10.1038/s41591-019-0620-2 31686035

[38] Wang C, Lv H, Ling X, . Association of assisted reproductive technology, germline de novo mutations and congenital heart defects in a prospective birth cohort study. Cell Res. 2021;31(8):919-928. doi:10.1038/s41422-021-00521-w 34108666 PMC8324888

[39] Sasani TA, Pedersen BS, Gao Z, . Large, three-generation human families reveal post-zygotic mosaicism and variability in germline mutation accumulation. Elife. 2019;8:e46922. doi:10.7554/eLife.46922 31549960 PMC6759356

[40] Mohiuddin M, Kooy RF, Pearson CE. De novo mutations, genetic mosaicism and human disease. Front Genet. 2022;13:983668. doi:10.3389/fgene.2022.983668 36226191 PMC9550265

[41] Vadlamudi L, Dibbens LM, Lawrence KM, . Timing of de novo mutagenesis—a twin study of sodium-channel mutations. N Engl J Med. 2010;363(14):1335-1340. doi:10.1056/NEJMoa0910752 20879882

[42] Yuan S, Guo L, Cheng D, . The de novo aberration rate of prenatal karyotype was comparable between 1496 fetuses conceived via IVF/ICSI and 1396 fetuses from natural conception. J Assist Reprod Genet. 2022;39(7):1683-1689. doi:10.1007/s10815-022-02500-5 35616756 PMC9365907

[43] Petrovski S, Aggarwal V, Giordano JL, . Whole-exome sequencing in the evaluation of fetal structural anomalies: a prospective cohort study. Lancet. 2019;393(10173):758-767. doi:10.1016/S0140-6736(18)32042-7 30712878

[44] Lord J, McMullan DJ, Eberhardt RY, ; Prenatal Assessment of Genomes and Exomes Consortium. Prenatal exome sequencing analysis in fetal structural anomalies detected by ultrasonography (PAGE): a cohort study. Lancet. 2019;393(10173):747-757. doi:10.1016/S0140-6736(18)31940-8 30712880 PMC6386638

[45] Jónsson H, Sulem P, Kehr B, . Parental influence on human germline de novo mutations in 1,548 trios from Iceland. Nature. 2017;549(7673):519-522. doi:10.1038/nature24018 28959963

[46] Kong A, Frigge ML, Masson G, . Rate of de novo mutations and the importance of father’s age to disease risk. Nature. 2012;488(7412):471-475. doi:10.1038/nature11396 22914163 PMC3548427

[47] García-Ferreyra J, Hilario R, Dueñas J. High percentages of embryos with 21, 18 or 13 trisomy are related to advanced paternal age in donor egg cycles. JBRA Assist Reprod. 2018;22(1):26-34. doi:10.5935/1518-0557.20180004 29303233 PMC5844656

[48] Duncan FE, Hornick JE, Lampson MA, Schultz RM, Shea LD, Woodruff TK. Chromosome cohesion decreases in human eggs with advanced maternal age. Aging Cell. 2012;11(6):1121-1124. doi:10.1111/j.1474-9726.2012.00866.x 22823533 PMC3491123

[49] Mazzilli R, Cimadomo D, Vaiarelli A, . Effect of the male factor on the clinical outcome of intracytoplasmic sperm injection combined with preimplantation aneuploidy testing: observational longitudinal cohort study of 1,219 consecutive cycles. Fertil Steril. 2017;108(6):961-972. doi:10.1016/j.fertnstert.2017.08.033 28985908

[50] Sartorius GA, Nieschlag E. Paternal age and reproduction. Hum Reprod Update. 2010;16(1):65-79. doi:10.1093/humupd/dmp027 19696093

