# Supplementary material for: Comparison of Genetic Profiles of Neonates in Intensive Care Units Conceived With or Without Assisted Reproductive Technology
Source: JAMA Netw Open. 2023 Apr 4;6(4):e236537. doi: 10.1001/jamanetworkopen.2023.6537 (PMC10074225; doi:10.1001/jamanetworkopen.2023.6537)
Supplement: Supplement 1. — eTable 1. The Ranking of Involved Organ Systems of the ART Group and Non-ART Group eTable 2. The Detailed Genetic Information of the 54 Diagnosed ART Cases eFigure. Distributions of Copy Number Variations (CNVs) and Single Nucleotide Variants (SNVs) in ART Offspring [file jamanetwopen-e236537-s001.pdf]

# Supplementary Online Content

Huang Z, Xiao F, Xiao H, et al. Comparison of genetic profiles of neonates in intensive care units conceived with or without assisted reproductive technology. *JAMA Netw Open.* 2023;6(4):e236537. doi:10.1001/jamanetworkopen.2023.6537

**eTable 1.** The Ranking of Involved Organ Systems of the ART Group and Non-ART Group
**eTable 2.** The Detailed Genetic Information of the 54 Diagnosed ART Cases
**eFigure.** Distributions of Copy Number Variations (CNVs) and Single Nucleotide Variants (SNVs) in ART Offspring

This supplementary material has been provided by the authors to give readers additional information about their work.

© 2023 Huang Z et al. *JAMA Network Open.*

**eTable 1. The Ranking of Involved Organ Systems of the ART Group and Non-ART Group**

| Rank* | Organ system | ART group | | Non-ART group | |
|---|---|---|---|---|---|
| | | All patients (n=535), No. (%) | Diagnosed patients (n=54), No. (%) | All patients (n=1316), No. (%) | Diagnosed patients (n=174), No. (%) |
| 1 | Cardiovascular** | 363 (67.9) | 25 (46.3) | 400 (30.4) | 49 (28.2) |
| 2 | Hepatic | 338 (63.1) | 20 (37.0) | 671 (51.0) | 35 (20.1) |
| 3 | Respiratory | 322 (60.2) | 28 (51.9) | 443 (33.7) | 34 (19.5) |
| 4 | Neuromuscular | 221 (41.3) | 34 (63.0) | 699 (53.1) | 75 (43.1) |
| 5 | Allergy/Immunologic/infectious | 209 (39.0) | 11 (10.4) | 526 (40.0) | 44 (25.3) |
| 6 | Hematologic | 207 (38.6) | 14 (25.9) | 597 (45.4) | 51 (29.3) |
| 7 | Metabolic | 140 (26.1) | 17 (31.5) | 560 (42.6) | 39 (22.4) |
| 8 | Gastrointestinal | 104 (19.4) | 11 (20.4) | 290 (22.0) | 23 (13.2) |
| 9 | Carniofacial | 74 (13.8) | 15 (27.8) | 51 (3.9) | 13 (7.5) |
| 10 | Hearing | 57 (10.6) | 3 (5.6) | 113 (8.6) | 6 (3.4) |
| 11 | Renal/Genital | 52 (9.7) | 8 (14.8) | 103 (7.8) | 16 (9.2) |
| 12 | Dermatologic | 38 (7.1) | 7 (13.0) | 97 (7.4) | 18 (10.3) |
| 13 | Skeletal | 37 (6.9) | 7 (13.0) | 106 (8.1) | 5 (2.9) |

* The ranking was based on the clinical presentation of these patients, and one patient may involve more than one feature; the depth of the color indicates the order of ranking among the top 4 involved organ systems.
** patent foramen ovale, patent ductus arteriosus, atrial septal defect, ventricular septal defect, as well as severe features (eg. tetralogy of Fallot) were all counted with cardiovascular abnormalities, while the cardiovascular anomaly is ranked the 1$^{th}$ in the ART cases.

© 2023 Huang Z et al. *JAMA Network Open.*

**eTable 2. The Detailed Genetic Information of the 54 Diagnosed ART Cases**

| Case No. | Test method | Variation information | Chromosome location | Zygosity | Inheritance/Source | Disease [OMIM] |
|---|---|---|---|---|---|---|
| 1 | WES | KMT2D,NM_003482:exon39:c.10880del(p.L3627Rfs*31) | chr12:49427607 | Het | AD/*De novo* | Kabuki syndrome 1 [147920] |
| 2 | CES | KCNQ2,NM_172107:exon14:c.1601del(p.P534Rfs*31) | chr20:62045470 | Het | AD/Paternal | Early infantile epileptic encephalopathy 7 [613720] |
| 3 | CES | TSC1,NM_000368:exon15:c.1888_1891del(p.K630Qfs*22) | chr9:135781078_135781081 | Het | AD/*De novo* | Tuberous sclerosis-1 [191100]; Lymphangioleiomyomatosis [606690] |
| 4 | WES | DUOX2,NM_014080:exon20:c.2635G>A(p.E879K)<br>DUOX2,M_014080:exon25:c.3329G>A(p.R1110Q) | chr15:45396177 | Het | AR/ Maternal/Paternal | Thyroid dyshormonogenesis 6 [607200] |
| 5 | CES | F11,NM_000128:exon13:c.1489C>T(p.R497X) | chr4:187207577 | Het | AD/Paternal | Factor XI deficiency, autosomal dominant [612416] |
| 6 | CES | G6PD,NM_001042351:exon12:c.1376G>T(p.R459L) | chrX:153760484 | Hemi | XLD/*De novo* | Hemolytic anemia due to G6PD deficiency [300908] |
| 7 | CES | GCDH,NM_000159:exon6:c.416C>G(p.S139W)<br>GCDH,NM_000159:exon3:c.109_110del(p.Q37Efs*5) | chr19:13004378;<br>chr19:13002318_13002319 | Het | AR/ Maternal/Paternal | Glutaricaciduria, type I [231670] |
| 8 | CES | JAG1,NM_000214:exon6:c.863G>A(p.W288X) | chr20:10633139 | Het | AD/Maternal | Alagille syndrome 1 [118450] |
| 9 | CES | COL2A1 ,NM_001844:exon32:c.2050-1G>C | chr12:48376921 | Het | AD/*De novo* | Kniest dysplasia [156550] |
| 10 | CES | ANKRD11,NM_013275:exon9:c.4205dup(p.Y1402X) | chr16:89348744 | Het | AD/*De novo* | KBG syndrome [148050] |
| 11* | CES | PTPN11,NM_002834:exon13:c.1528C>G(p.Q510E) | chr12:112926908 | Het | AD/*De novo* | Noonan syndrome 1 [163950];<br>LEOPARD syndrome 1[151100];<br>Metachondromatosis,[156250] |
| 12* | CES | PTPN11,NM_002834:exon13:c.1528C>G(p.Q510E) | chr12:112926908 | Het | AD/*De novo* | Noonan syndrome 1,[163950];<br>LEOPARD syndrome 1[151100];<br>Metachondromatosis[156250] |
| 13 | CES | G6PD,NM_001042351:exon12:c.1388G>A(p.R463H) | chrX:153760472 | Hemi | XLD/Maternal | Hemolytic anemia, G6PD deficient (favism) [300908] |
| 14 | CES | COL7A1,NM_000094:c.3809C>T(p.P1270L)<br>COL7A1,NM_000094:c.4556G>A(p.G1519D)<br>COL7A1,NM_001042492:c.6854dup(p.Y2285X) | chr3:48623145 | Het | AR/ Maternal/Paternal | EBD inversa [226600];<br>Epidermolysis bullosa dystrophica, AR [226600] |

© 2023 Huang Z et al. *JAMA Network Open.*

| | | | | | | |
|---|---|---|---|---|---|---|
| 15 | CES | SCN2A,NM_021007:exon7:c.788C>T(p.A263V) | chr2:166166923 | Het | AD/*De novo* | Epileptic encephalopathy, early infantile, 11 [613721] |
| 16 | CES | KCNQ2,NM_172107:exon4:c.620G>A(p.R207Q) | chr20:62076082 | Het | AD/*De novo* | Seizures, benign neonatal, 1 [121200] |
| 17 | WES | ATP7A,NM_000052:exon17:c.3301G>A(p.D1101N) | chrX:77289109 | Hemi | XLR/*De novo* | Menkes disease [309400] |
| 18 | CES | ABCC8,NM_000352:exon25:c.2992C>T(p.R998X) | chr11:17428605 | Het | AD/*De novo* | Diabetes mellitus, transient neonatal 2 [610374] |
| 19 | CES | NSD1,NM_022455:exon7:c.4075del(p.S1359Qfs*13) | chr5:176665390 | Het | AD/Paternal | Sotos syndrome 1 [117550] |
| 20 | WES | RYR1,NM_000540:exon25:c.3224G>A(p.R1075Q) RYR1,NM_000540:exon4:c.325C>T(p.R109W) | chr19:38958295 | Het | AR/ Maternal/Paternal | Central core disease [117000] |
| 21* | CES | MAF,NM_005360:exon1:c.161C>T(p.S54L) | chr16:79633639 | Het | AD/*De novo* | Ayme-Gripp syndrome [601088] |
| 22* | CES | MAF,NM_005360:exon1:c.161C>T(p.S54L) | chr16:79633639 | Het | AD/*De novo* | Ayme-Gripp syndrome [601088] |
| 23 | CES | NF1,NM_000267:exon26:c.3447G>A(p.M1149I) | chr17:29559850 | Het | AD/*De novo* | Neurofibromatosis, type 1 [162200] |
| 24 | CES | MYH7,NM_000257:exon7:c.602T>C(p.I201T) | chr14:23901007 | Het | AD/Paternal | Cardiomyopathy, dilated, 1S [613426] |
| 25 | CES | MYH9,NM_002473:exon39:c.5521G>A(p.E1841K) | chr22:36680520 | Het | AD/Paternal | Macrothrombocytopenia and granulocyte inclusions with or without nephritis or sensorineural hearing loss [155100] |
| 26 | CES | RUNX2,NM_001024630:exon7:c.860-1G>C | chr6:45479982 | Het | AD/*De novo* | Cleidocranial dysplasia, forme fruste, with brachydactyly [119600] |
| 27 | CES | CAMK2B,NM_001220:exon7:c.416C>T(p.P139L) | chr7:44283125 | Het | AD/*De novo* | Mental retardation, autosomal dominant 54 [617799] |
| 28 | WES | USP9X,NM_001039590:exon31:c.4672G>A(p.G1558R) | chrX:41060381 | Het | XLD/*De novo* | Mental retardation, X-linked 99 [300919] |
| 29 | WES | DMD,NM_004006:exon13:c.1555G>T(p.E519X) | chrX:32613921 | Hemi | XLR/Maternal | Duchenne muscular dystrophy [310200] |
| 30 | CES | RAF1,NM_002880:exon14:c.1472C>G(p.T491R) | chr3:12627244 | Het | AD/*De novo* | Noonan syndrome 5 [611553] |
| 31 | CES | COL2A1,NM_001844:exon27:c.1780G>A(p.G594R) | chr12:48378831 | Het | AD/*De novo* | Achondrogenesis, type II or hypochondrogenesis [200610] |

© 2023 Huang Z et al. *JAMA Network Open.*

| | | | | | | |
|---|---|---|---|---|---|---|
| 32 | CES | CAMTA1,NM_015215:exon5:c.427C>T(p.Q143X) | chr1:7309675 | Het | AD/*De novo* | Cerebellar ataxia, nonprogressive, with mental retardation [614756] |
| 33 | CES | FGFR3,NM_000142:exon7:c.749C>G(p.P250R) | chr4:1803571 | Het | AD/Paternal | Hypochondroplasia [146000] |
| 34 | CES | BRAF,NM_004333:exon13:c.1574T>C(p.L525P) | chr7:140476832 | Het | AD/*De novo* | Noonan syndrome 7 [613706] |
| 35 | WES | 1q41-1q42.13 DEL | chr1:223116148_227175245 | . | *De novo* | Leukodystrophy, hypomyelinating, 19, transient infantile [618688] |
| 36 | WES | 13q22.3 DEL | chr13:77566178_78493903 | . | *De novo* | . |
| 37 | WES | aneuploidy | chr18:158382_78005429 | . | *De novo* | Edwards Syndrome; Trisomy 18 |
| 38 | CES | 7q11.23 DEL | chr7:73442118_74148323 | . | *De novo* | . |
| | | 22q11.23 DUP | chr22:23656154_24922636 | . | *De novo* | . |
| 39 | WES | Xq22.2 DUP | chrX:102831158_103268259 | . | *De novo* | Pelizaeus-Merzbacher disease [312080] |
| 40 | CES | 14q31.3-14q32.2 DEL | chr14:88852000_97347946 | . | *De novo* | . |
| 41 | CES | 10q23.2-10q23.31 DEL | chr10:88809958_91222381 | . | *De novo* | Multisystemic smooth muscle dysfunction syndrome [613834] |
| 42 | WES | 4p16.3 DEL | chr4:53197_3534286 | . | *De novo* | Wolf-Hirschhorn syndrome [194190] |
| 43 | CES | 19p13.2 DEL | chr19:13135394_13482593 | . | *De novo* | . |
| 44 | CES | 17p12 DUP | chr17:14110126_15492541 | . | *De novo* | Dejerine-Sottas disease [145900] |
| 45 | CES | 22q11.21 DEL | chr22:18893735_20307603 | . | *De novo* | DiGeorge syndrome [188400] |
| 46 | CES | 1p21.1 DEL | chr1:102271631_104162406 | . | *De novo* | . |
| 47 | WES | 3p14.2-3p14.1DEL | chr3:59737132_66024225 | . | *De novo* | Spinocerebellar ataxia 7 [164500] |
| 48 | WES | Xq28 DUP | chrX:153030925_153599729 | . | *De novo* | Syndromic X-linked intellectual disability Lubs type |
| 49 | CES | aneuploidy (47, XXY) | chrX:2700106_22269427 | . | *De novo* | Klinefelter syndrome |
| 50 | WES | 15q11.2-15q13.2 DUP | chr15:22070539_30702470 | . | *De novo* | Prader-Willi syndrome [176270] |
| 51 | WES | Xp21.1 DEL | chrX:31947712_31986631 | . | *De novo* | Duchenne muscular dystrophy [310200] |

© 2023 Huang Z et al. *JAMA Network Open.*

| | | | | | | |
|---|---|---|---|---|---|---|
| 52 | CES | 10q24.1-10q26.2 DUP | chr10:98703869_129924020 | . | *De novo* | . |
| 53 | CES | 4p16.3 DEL | chr4:85621_3770253 | . | *De novo* | Wolf-Hirschhorn syndrome [194190] |
| 54 | WES | 22q11.21 DEL | chr22:18893832_21563415 | . | *De novo* | DiGeorge syndrome [188400] |

Abbreviations: CES, clinical exome sequencing; WES, whole exome sequencing; Het, heterozygote; Hemi, hemizygote; AD, autosomal dominant; AR, autosomal recessive; XLD, X-linked dominant; XLR, X-linked recessive; DEL, deletion; DUP, duplication

* Case 11 and 12; case 21 and 22 were twins, respectively.

© 2023 Huang Z et al. *JAMA Network Open.*

**eFigure**. Distributions of Copy Number Variations (CNVs) and Single Nucleotide Variants (SNVs) in ART Offspring

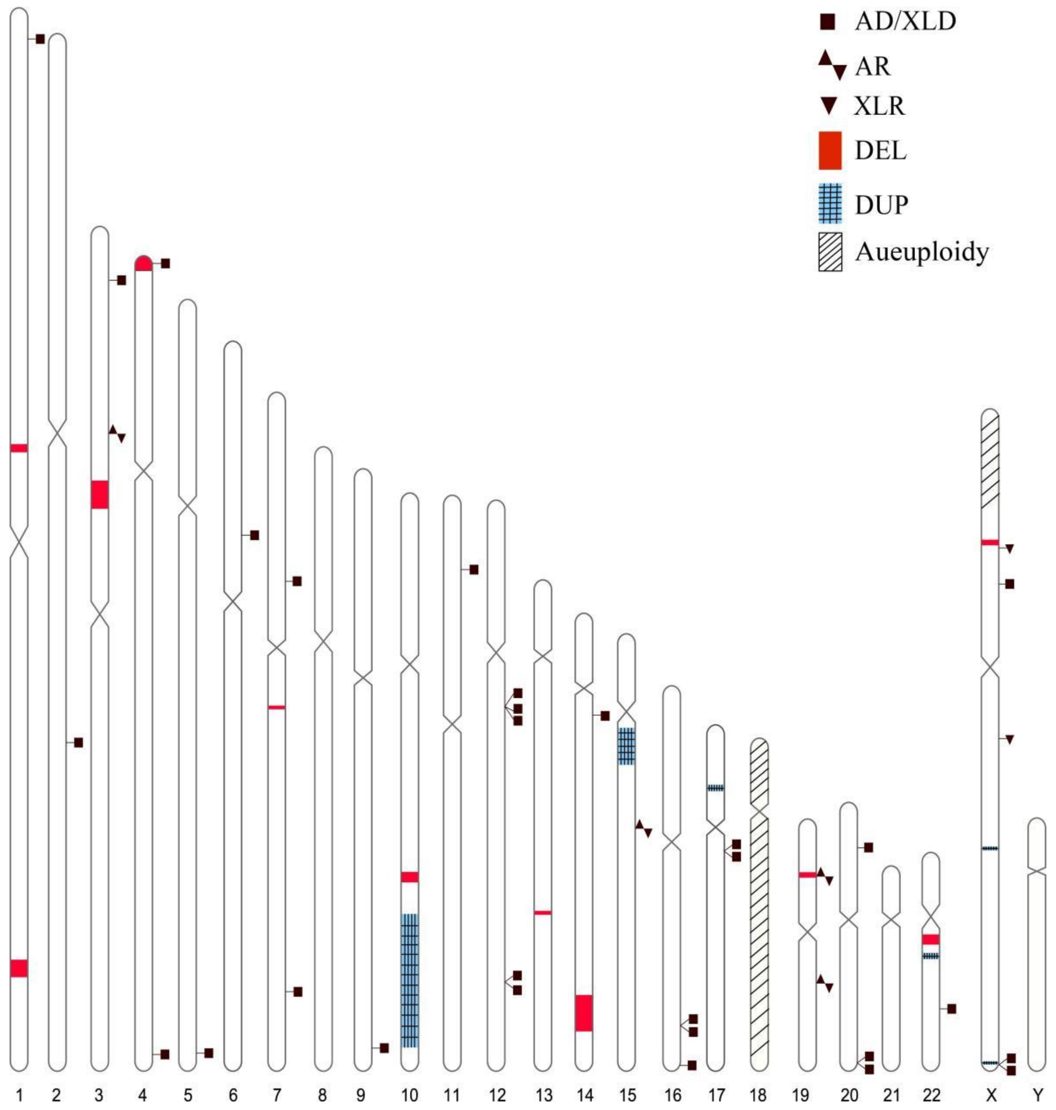


Dominantly inherited SNVs are represented by squares while recessively inherited SNVs are represented by triangles. CNV deletion and duplication is shown in red and blue with lines respectively. The length of the bars indicates the size of each loss or gain region plotted on the chromosome scale. Slashes represent aneuploidy, as shown in chromosomes 18, 21.

© 2023 Huang Z et al. *JAMA Network Open.*
