# Supplementary material for: Comparison of Genetic Profiles of Neonates in Intensive Care Units Conceived With or Without Assisted Reproductive Technology
Source: JAMA Netw Open. 2023 Apr 4;6(4):e236537. doi: 10.1001/jamanetworkopen.2023.6537 (PMC10074225; doi:10.1001/jamanetworkopen.2023.6537)
Supplement: Supplement 2. — Data Sharing Statement [file jamanetwopen-e236537-s002.pdf]

Data Sharing Statement

Huang. Comparison of Genetic Profiles of Neonates in Intensive Care Units Conceived With or Without Assisted Reproductive Technology. *JAMA Netw Open*. Published April 04, 2023. doi:10.1001/jamanetworkopen.2023.6537

Data
Data available: Yes
Data types: Deidentified participant data
How to access data: Emails could be sent to the address below to obtain the shared data: huijunwang@fudan.edu.cn.
When available: With publication

Supporting Documents
Document types: None

Additional Information
Who can access the data: Researchers whose proposed use of the data has been approved.
Types of analyses: For analysis of the genetic profile of ART offspring.
Mechanisms of data availability: With investigator support.
Any additional restrictions: We may balance the potential benefits and risks for each request and then provide the data that could be shared.
